# Alterations in Electroencephalography Theta as Candidate Biomarkers of Acute Cannabis Intoxication

**DOI:** 10.3389/fnins.2021.744762

**Published:** 2021-10-04

**Authors:** Christian D. Richard, Jared R. Poole, Marissa McConnell, Amir H. Meghdadi, Marija Stevanovic-Karic, Greg Rupp, Abigail Fink, Rose Schmitt, Timothy L. Brown, Chris Berka

**Affiliations:** ^1^Advanced Brain Monitoring, Carlsbad, CA, United States; ^2^National Advanced Driving Simulator, The University of Iowa, Iowa City, IA, United States

**Keywords:** electroencephalography, event-related potentials, cannabis, biomarkers, attention, memory, intoxication

## Abstract

The trend toward cannabis legalization in the United States over the past two decades has unsurprisingly been accompanied by an increase in the number of cannabis users and use patterns that potentially pose wider risks to the public like driving under the influence. As such, it is becoming increasingly important to develop methods to accurately quantify cannabis intoxication and its associated impairments on cognitive and motor function. Electroencephalography (EEG) offers a non-invasive method for quantitatively assessing neurophysiological biomarkers of intoxication and impairment with a high degree of temporal resolution. Twelve healthy, young recreational cannabis users completed a series of neurocognitive tasks with concurrent EEG acquisition using the ABM STAT X24 EEG headset in a within-subject counterbalanced design. The 1-h testbed consisted of resting state tasks and tests of attention and memory. Spectral densities were computed for resting state tasks, and event-related potentials (ERPs) were obtained for the attention and memory tasks. Theta band power (3–5 Hz) was decreased during cannabis intoxication compared to placebo during resting state tasks, as were average P400 and late positive potential (LPP) amplitudes during attention and memory tasks. Cannabis intoxication was also associated with elevated frontal coherence and diminished anterior–posterior coherence in the Theta frequency band. This work highlights the utility of EEG to identify and quantify neurophysiological biomarkers from recordings obtained during a short neurocognitive testbed as a method for profiling cannabis intoxication. These biomarkers may prove efficacious in distinguishing intoxicated from non-intoxicated individuals in lab and real-world settings.

## Introduction

The decades long trend toward decriminalization and legalization of cannabis in the United States has accelerated in recent years. As of 2020, 15 states have fully legalized cannabis, 22 states have legalized medical cannabis, and 14 states have implemented limited medical cannabis laws that only permit use of cannabidiol (CBD) (Yu et al., 2020). At the Federal level, cannabis is currently classified as a Schedule I Drug, defined as having a high potential for abuse without any medicinal value, despite biomedical research unequivocally demonstrating efficacy in treatment of Dravet and Lennox–Gastaut epileptic syndromes (Thiele et al., 2018; Chen et al., 2019). The bureaucratic hurdles erected against research on cannabis discourage the investigations required to clearly delineate its short- and long-term risks and benefits (Yang and Szaflarski, 2019).

This need for more research is no less true for studies attempting to quantify the biological correlates of cannabis intoxication and impairment, especially when considering the potential for increases in motor vehicle accidents by drivers under the influence of cannabis (Compton, 2017). As cannabis use becomes more widely accepted, there is growing interest in its effects on brain function, specifically how it may impact daily functional activities such as driving, operating machinery and other safety-related tasks. From a public safety standpoint it is essential for employers, policymakers, and law enforcement officials to fully understand the effects of cannabis on all aspects of performance, and to do so requires the identification of biomarkers specific to acute cannabis intoxication.

Unlike the intoxicating effects of alcohol which can be reliably assessed by blood alcohol concentrations, a determination of cannabis intoxication and impairment can’t be based solely on blood concentrations of delta-9-tetrahydrocannabinol (Δ^9^-THC) or its metabolites due to their persistence in body tissues long after the intoxicating effects have passed (Neavyn et al., 2014). However, a growing number of human studies have added to the list of potential biomarkers by employing electroencephalography (EEG) to probe the neuroelectric activity underlying the psychoactive effects of cannabis.

The cannabis plant contains over 500 phytochemical compounds (Mechoulam, 2005; Appendino et al., 2011; Pertwee, 2014; Andre et al., 2016), with the two main constituents being Δ^9^-THC and CBD. There are two known cannabinoid receptors: type 1 (CB1) and type 2 (CB2). These receptors interact with the circulating endogenous cannabinoids aiding in the regulation of stress response, emotion, neuroplasticity, and homeostasis (Egerton et al., 2006; Befort, 2015). Δ^9^-THC exerts its psychoactive effects through interactions with CB1 receptors in the central nervous system with high receptor densities in the prefrontal cortex, hippocampus, basal ganglia, and cerebellum (Burns et al., 2007; Lorenzetti et al., 2016; Hillard, 2018). CBD has also been shown to bind to CB1 receptors, mitigating some of the psychoactive effects of THC (Laprairie et al., 2015; Lorenzetti et al., 2016).

The objective of this study was to identify candidate biomarkers that could be used as a quantitative measures of acute cannabis intoxication and impairment to facilitate the development of a Cannabis Impairment Detection Application (CIDA), a platform employing time-synchronized acquisition of EEG, electrocardiogram (ECG), and performance data from tests shown to be sensitive to acute cannabis intoxication. To accomplish this, we designed and implemented a double-blinded placebo controlled cross-over study to assess the effects of acute cannabis intoxication on EEG and ECG acquired during resting state conditions, and event-related potential (ERPs) during engagement with neurocognitive tests of attention and memory.

## Materials and Methods

### Subjects

Fourteen subjects (six females and eight males) participated in both the Δ^9^-THC and placebo visits. The average age in years of subjects was 24.1 ± 1.4, ranging from 19 to 34 years old. Self-reported cannabis usage ranged from an annual 5–6 times to 208 times a year (4 times a week).

### Cannabis

The active cannabis used for the dosing session contained 6.7% Δ^9^-THC by weight and the placebo cannabis used for the placebo session contained 0.009% Δ^9^-THC. The active cannabis dose was chosen to ensure that subjects were intoxicated. Average Δ^9^-THC concentrations in cannabis available today can range from 5 to 15% with some strains reaching as high as 25–30% (Peters and Chien, 2018).

During the experimental sessions, the subject was administered 500mg of either active or placebo cannabis using the Volcano Digit Vaporizer (Storz & Bickel, GmbH, Germany). Apart from the differences in Δ^9^-THC concentrations, the active and placebo cannabis were otherwise indistinguishable. Cannabis was obtained from NIDA Drug Supply Program.

### Electroencephalography and Electrocardiogram Recordings

Electroencephalography and ECG were acquired using the STAT X24 wireless sensor headset (Advanced Brain Monitoring, Inc., Carlsbad, CA, United States). This system has 20-channels of EEG located according to the International 10–20 system and an auxiliary channel for ECG. Linked reference electrodes were located behind each ear on the mastoid bone. Each sensor site was covered with a single-use foam pad filled with an electrolyte infused cream (Synapse, Kustomer Kinetics) which improves contact with the scalp resulting in higher conductivity. Impedance measurements taken at each electrode were deemed to be within ideal tolerance at or below 40 kΩ which are within the tolerance specifications of the EEG hardware utilized for the study. Sigma-delta 16-bit A/D conversion and amplification are done at electrode sites permitting high fidelity data capture at greater impedance cut-offs.

Heart activity was gathered with ECG from electrodes situated on the left and right clavicles. The ECG signal is first filtered to improve the contrast between the QRS complex and the T wave. The inter-beat R–R interval represents the number of seconds between consecutive R-waves. Heart rate is estimated as a number of beats per minute, i.e., 60/R–R interval. The algorithm assesses the quality of detected beats by monitoring the standard deviation of the consecutive beats. Heart rate variability was also computed to quantify parasympathetic vs. sympathetic arousal, evaluated through the ratio of low frequency (LF) to high frequency (HF) HRV. Higher LF/HF ratios are associated with increased sympathetic activation, and stress/anxiety states (Prashad et al., 2018).

Sampling rate was 256 Hz with high band pass at 0.1 Hz and a low band, fifth order filter, at 100 Hz. Physiological data were transmitted wirelessly *via* Bluetooth to a host computer for storage.

### Cannabis Impairment Detection Application Testbed

The CIDA platform is comprised of a series of neurocognitive tasks that test attention and verbal memory with concurrent EEG and ECG data acquisition. Tasks were administered on a portable computing device (iPad, Apple Inc., Cupertino, CA, United States) to be suitable for proposed field deployment.

#### Resting State Eyes Open and Resting State Eyes Closed

**FIGURE 1:**
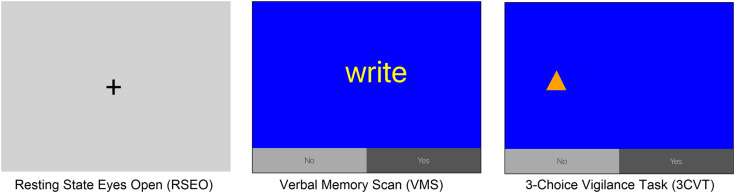
Display screens of task interfaces for resting state, VMS, and 3CVT. Left panel: fixation cross for RSEO; middle panel: VMS; and right panel: 3CVT.

Resting state eyes open (RSEO) is a 5-min task where the subject is asked to stare at a cross at the center of the screen (Figure 1, left panel). The subject is instructed to sit as still as possible while trying to refrain from excessive eye blinks. Resting state eyes closed (RSEC) is similar except the subject is asked to close his or her eyes and sit as still as possible.

#### Verbal Memory Scan Task

The 20-min verbal memory scan (VMS) is a Sternberg serial probe recognition memory task developed to measure speed and accuracy of verbal working memory. Lists of five words (memory sets) are presented one word at a time, followed by single-word probes (in-set or out-of-set). Subjects click the “Yes” button if the probe word was in-set or “No” if out-of-set (Figure 1, middle panel). Fifty percent of probes are “Yes” (Target) and 50% are “No” (NonTarget) probe words.

#### Three-Choice Vigilance Task

The three-choice vigilance task (3CVT) incorporates features from the most common measures of sustained attention, including the Continuous Performance Test, Wilkinson Reaction Time, and the PVT-192 (Berka et al., 2005). The 3CVT takes about 20-min to complete and requires discrimination between a Target geometric shape and two NonTarget ones presented for 0.2 s at a time (Figure 1, right panel). This task presents three different shape stimuli: right-side-up triangles (▲), upside-down triangles (▼), and diamonds (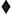). Right-side-up triangles are presented 70% of the time, diamonds presented 15% of the time, and the upside-down-triangles are presented the remaining 15% of the time at varying inter-stimulus time intervals from 1.5 to 10 s. A training-to-criterion practice session was provided prior to the start to make sure subjects could follow instructions and to minimize practice effects. Subjects select the “Yes” button for the right-side-up triangle (Target stimulus) and the “No” button for the other two shapes (NonTarget stimuli).

### Protocol

Eligible subjects completed a total of three visits: a screening and two experimental visits. After informed consent, the screening visit consisted of an initial urinalysis to test for presence of illicit drugs and pregnancy, a brief physical examination of vital signs including heart rate and blood pressure, and a psychiatric exam. After successful completion of the physical and psychiatric screenings, an in-depth survey was administered that included detailed demographics and questions about the presence and extent of any preexisting abnormalities and/or mental health issues that may put the participant at a greater risk for health complications, adverse drug reactions, or interfere with the study procedures and results.

Upon arrival for the experimental visits, staff collected another urine sample to test for illicit drug use and pregnancy. Subjects then completed a sleep and food intake questionnaire (Sleep and Food Intake Survey) and had to show that they had 7–9 h of sleep the night before the study visit. They then filled out a questionnaire about their current sleepiness level (Stanford Sleepiness Scale). If the subject met the study criteria, they were fitted with the STAT X24 EEG Wireless Sensor Headset. Subjects were then escorted to a private room and were administered 500 mg of either active cannabis or a placebo cannabis *via* inhalation using a Volcano Digit Vaporizer, followed by rest for 10 min. After this rest period, subjects provided self-assessments of a set of descriptors capturing the psychological and physical symptoms associated with cannabis intoxication (Good Drug Effect, High, Stoned, Stimulated, Sedated, Anxious, and Restless). Descriptors were rated along 0–100 range anchored with “not at all” and “most ever,” respectively. Subjects then completed an approximately 60-min CIDA Assessment, which is described above.

All potential enrollees were then asked to complete a brief (5–8 min) drive in a driving simulator (Brown et al., 2019, 2020). Following the drive, subjects were administered a wellness questionnaire to gauge their risk of simulator sickness based on scores for nausea, oculomotor effects, and disorientation. All subjects passed and were allowed to continue with the study. The experimental sessions were identical except for whether the subject was administered active cannabis (THC visit) or placebo (Placebo visit) the order of which were counterbalanced. All protocols and procedures were approved by the University of Iowa IRB.

The date of the second experimental session was confirmed at the end of the first visit. This next visit was scheduled at least 1 week after the first one took place in order to ensure that any potential drug used in the study had washed out of the participant’s system before the start of the next visit. After each visit, subjects were provided transportation home to ensure they were not driving under any potential influence due to the study procedures.

### Electroencephalography Data Analysis

#### Spectral Density

Data were bandpass filtered (1–40 Hz). Independent component analysis (ICA) and artifact decontamination was performed using ICLabel toolbox to reject components classified as having sources other than brain (e.g., eye blinks, EMG, etc.). ICLabel uses a classifier that is pre-trained by thousands of labeled components obtained through crowdsourcing (Pion-Tonachini et al., 2019). Power spectral densities (PSDs) were then computed for each 1 s epoch by averaging Fast Fourier Transforms of the EEG segment corresponding to each epoch along with the two EEG segments immediately preceding and following that share 50% overlap with the given epoch. Kaiser window was applied to the EEG segments prior to Fast Fourier Transform. The total power in each frequency bin 1–40 Hz and eight (8) frequency bandwidths were computed for each 1-s epoch. The frequency bands were defined as Delta (1–3 Hz), slow Theta (3–5 Hz), fast Theta (5–7 Hz), slow Alpha (8–10 Hz), fast Alpha (10–12 Hz), slow Beta (13–20 Hz), fast Beta (21–30 Hz), and Gamma (30–40 Hz). PSD analyses were completed for RSEO and RSEC.

#### Event-Related Potentials

Electroencephalography data were time-synchronized with stimuli and responses during the 3CVT and VMS allowing for ERPs to be computed and measured. For 3CVT and VMS, raw EEG signals were filtered between 0.1 and 40 Hz using a Hamming windowed Sinc FIR filter (8449-point filtering with a 0.1 Hz transition band width). For each event type, EEG data were parsed into epochs from 1000 ms before the stimulus onset until 2000 ms after stimulus presentation. The P200, P400, and late positive potential (LPP) ERP components were assessed in both 3CVT and VMS tasks. Amplitudes of P200 and P400 peaks were measured within a time window from +150 to +250 ms, and +275 to +475 ms post-stimulus presentation, respectively. Measurements of the LPP component were taken from +500 to +800 ms. The baseline was adjusted using data from 100 ms before the stimulus onset.

#### Inter-Channel Coherence

The main method for coherence analysis was magnitude-squared coherence based on the mscohere function in MATLAB (MathWorks Inc., Natick, MA, United States). Estimates of magnitude-squared coherence were calculated as a function of frequency *f* between pairs of EEG channels x and y from:


$$C_{x\,y}\,\left(f\right)=\frac{{\left|P_{x\,y}\,\left(f\right)\right|}^{2}}{P_{x\,x}\,\left(f\right)\,P_{y\,y}\,\left(f\right)}$$

where P_*xy*_(f) is the cross power spectral density function between ICLabel-filtered EEG signals x and y computed using Welch’s overlapped averaged periodogram with a moving average 2-s Kaiser window set for 50% overlap. We compute C_*xy*_(f) for each 20-s analysis window separately and report the average and standard error of coherence on a 20-s analysis window.

#### Data Exclusion

Electroencephalography trials with spectrum 35 dB higher or lower than the baseline in the frequency range of 20–30 Hz were excluded. A minimum of 24 clean epochs in EEG data, and at least 12 good trials from ERP data were required for inclusion in analyses. ERP trials were rejected if the absolute value of EEG amplitude in any channel during a window of −50 to +750 ms, relative to the stimulus onset, exceeded a threshold level of 100 μV. Trials with high kurtosis or low probability of occurrence were excluded using a threshold of 6 *z*-score per component and 5 *z*-score per average of components. The within-subject design necessitated that any subjects missing either visit as a result of data exclusion were not included in subsequent analyses. Four subjects (two males and two female) were excluded from resting state analysis due to withdrawal after their first visit; ERP data from seven subjects were excluded (three females and four males) because of missed visits or not meeting minimum number of useable ERP trials.

#### Statistical Analysis

Channelwise analyses of EEG power from RSEO and RSEC, and the ERP components P200, P400, and LPP amplitudes from 3CVT and VMS tasks, were conducted using paired *t*-tests to assess within-subject effects of dose (THC and Placebo visits), and stimulus type for ERP measures (Target and NonTarget stimuli). Self-reports of intoxication measures from the cannabis and placebo visits were compared using paired *t*-tests. All hypothesis tests used two-tailed statistics. Statistical analyses were conducted using MATLAB. The α criterion for significance was set to 0.05. False discovery rate (FDR) was employed to correct for multiple comparisons (Benjamini and Hochberg, 1995). Significant results before and after application of FDR are both reported.

## Results

### Intoxication Measures

Self-reported ratings for descriptors commonly associated with cannabis intoxication were in alignment with the actual dose they received. These subjective assessments, on a scale from 0 to 100, were taken shortly after inhalation of either cannabis or placebo to guarantee that those receiving cannabis would be in an acute stage of intoxication. The greatest disparities in intoxication ratings between the two visits were found for the descriptors “High” (*p* = 0.000001, *t*: 11.4; THC visit: 70.1 ± 2.1, Placebo visit: 7.9 ± 1.0) and “Stoned” (*p* = 0.00007, *t*: 6.9; THC visit: 63.2 ± 3.0, Placebo visit: 4.0 ± 0.6).

While the magnitude of the differences between the two visits were not as great for the other descriptors, all were significant. “Good Drug Effect” (*p* = 0.00004, *t*: 7.4; THC visit: 67.7 ± 2.3, Placebo visit: 11 ± 1.3), “Stimulated” (*p* = 0.0018, *t*: 4.4; THC visit: 69.3 ± 2.2, Placebo visit: 22.6 ± 3.2), “Sedated” (*p* = 0.0002, *t*: 5.9; THC visit: 52.5 ± 3.1, Placebo visit: 7.8 ± 1.2), “Anxious” (*p* = 0.0003, *t*: 5.6; THC visit: 53.1 ± 2.8, Placebo visit: 3.2 ± 0.6), and “Restless” (*p* = 0.008, *t*: 3.4; THC visit: 37.5 ± 3.0, Placebo visit: 9.9 ± 1.9). Ratings by visit represent mean ± SEM, d.f. = 9 for all comparisons.

### Resting State Electroencephalography Data

**FIGURE 2:**
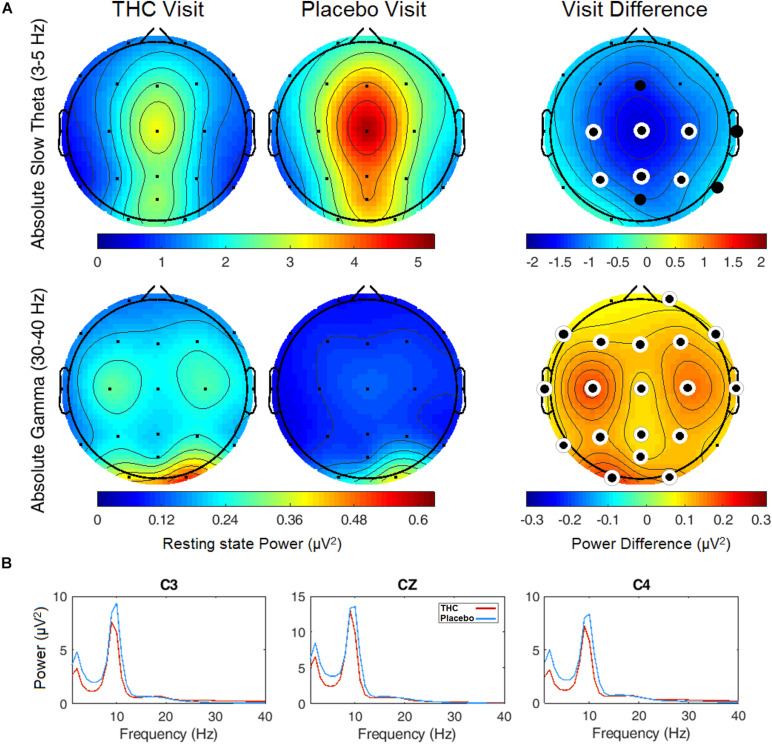
Resting state eyes closed by visit. THC visit associated with spectral power decreases at slow frequencies and increases at fast frequencies. **(A)** Topographic maps where significant differences after FDR correction for multiple comparisons are marked with white rings. Visit differences at slow Theta were significant across central and parietal channels (top row). Gamma band spectral power were greater in all channels but Fp1 and T6 for subjects during their THC visit (bottom row). **(B)** Power spectral densities at central channels. THC visit, red line; Placebo visit, blue line.

Resting state results for both eyes open and eyes closed conditions revealed decreases in spectral power at the slow Theta frequency band during the THC visit compared to the placebo visit. The effect of cannabis intoxication on slow Theta power was most pronounced in the RSEC condition (Figure 2A, slow Theta). The difference in spectral power between the two visits was greatest in central EEG channels. Although comparison of the two visit shows that the most dramatic changes were along the midline, we found evidence of widespread decreases in power particularly at slower frequency bands in the central channels (Figure 2B). The THC visit was associated with elevated power at higher frequencies, specifically in the Gamma frequency band range in right and left central EEG channels (Table 1).

**TABLE 1:** Paired *t*-test statistics for absolute spectral power during RSEC comparing the Δ9-THC and placebo condition.

Frequency band	Channel	*t*−statistic	d.f.	*p*-value	THC visit	Placebo visit	CI (lower, upper)
Slow Theta (3–5 Hz)	C3	–3.63	9	0.0055	1.544 ± 0.15	2.736 ± 0.18	−2.25, −0.13^‡^
	C4	–3.51	9	0.0066	1.558 ± 0.17	2.853 ± 0.22	−2.48, −0.18^‡^
	Cz	–4.27	9	0.0021	3.186 ± 0.32	4.996 ± 0.29	−3.18, −0.44^‡^
	Fz	–2.58	9	0.0295	2.478 ± 0.91	3.949 ± 1.46	−2.76, −0.18
	P3	–3.22	9	0.0105	1.473 ± 0.17	2.467 ± 0.2	−1.99, −1.6e−15^‡^
	P4	–3.28	9	0.0096	1.682 ± 0.22	2.734 ± 0.21	−2.09, −0.02^‡^
	POz	–2.82	9	0.02	2.728 ± 1.73	3.833 ± 1.47	−1.99, −0.22
	Pz	–3.48	9	0.0069	2.546 ± 0.29	3.925 ± 0.29	−2.65, −0.1^‡^
	T4	–2.4	9	0.04	0.946 ± 0.59	1.662 ± 0.78	−1.39, −0.04
	T6	–2.68	9	0.0253	0.825 ± 0.7	1.426 ± 0.75	−1.11, −0.09
Gamma (30–40 Hz)	C3	4.21	9	0.0023	0.291 ± 0.03	0.1 ± 0.01	0.085, 0.297^‡^
	C4	3.83	9	0.004	0.278 ± 0.03	0.115 ± 0.01	0.064, 0.262^‡^
	Cz	2.77	9	0.0217	0.225 ± 0.03	0.133 ± 0.01	0.015, 0.17^‡^
	F3	2.86	9	0.0188	0.207 ± 0.02	0.102 ± 0.01	0.02, 0.19^‡^
	F4	3.04	9	0.014	0.234 ± 0.03	0.11 ± 0.01	0.029, 0.22^‡^
	F7	2.35	9	0.0435	0.159 ± 0.02	0.078 ± 0.01	0.001, 0.161^‡^
	F8	2.36	9	0.0425	0.175 ± 0.03	0.089 ± 0.01	0.001, 0.17^‡^
	Fp2	2.4	9	0.0398	0.159 ± 0.02	0.082 ± 0.01	0.002, 0.151^‡^
	Fz	3.21	9	0.0107	0.211 ± 0.02	0.104 ± 0.01	0.029, 0.184^‡^
	O1	2.77	9	0.0216	0.394 ± 0.06	0.2 ± 0.04	0.031, 0.356^‡^
	O2	2.77	9	0.0218	0.508 ± 0.17	0.372 ± 0.16	0.022, 0.249^‡^
	P3	3.38	9	0.0082	0.228 ± 0.03	0.099 ± 0.01	0.04, 0.217^‡^
	P4	2.9	9	0.0177	0.251 ± 0.04	0.133 ± 0.02	0.023, 0.214^‡^
	POz	2.8	9	0.0206	0.239 ± 0.03	0.136 ± 0.02	0.018, 0.189^‡^
	Pz	2.79	9	0.0212	0.204 ± 0.03	0.114 ± 0.01	0.015, 0.167^‡^
	T3	2.33	9	0.045	0.135 ± 0.02	0.058 ± 0.01	0, 0.153^‡^
	T4	2.89	9	0.0179	0.211 ± 0.03	0.105 ± 0.02	0.021, 0.192^‡^
	T5	3.12	9	0.0123	0.166 ± 0.02	0.065 ± 0.01	0.026, 0.176^‡^

*^‡^Significant after FDR correction for multiple comparisons; d.f., degrees of freedom; CI, confidence interval.*

**FIGURE 3:**
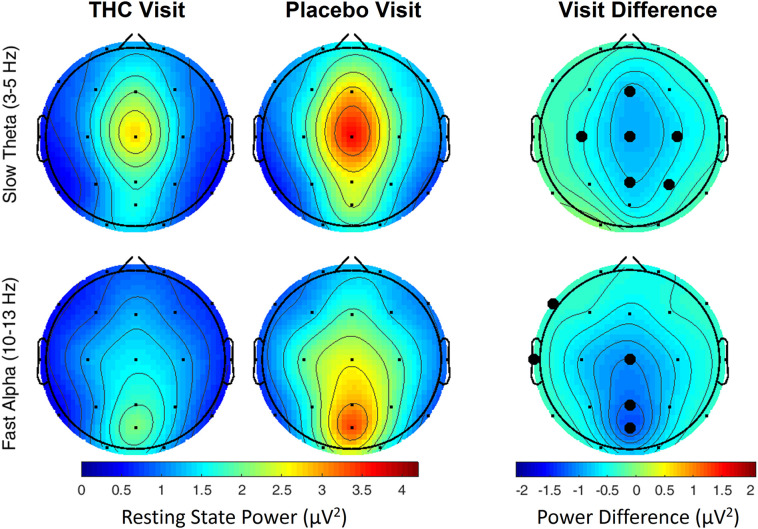
Resting state eyes open by visit. During THC visit, spectral power fell relative to placebo visit at slow Theta and fast Alpha frequency bands. Topographic maps show widespread decreases in midline Theta (top row) and fast Alpha (bottom row) from THC visit to placebo visit. Filled black circles mark channels that were significantly different between the visits prior to FDR correction for multiple comparisons.

The effects of cannabis intoxication on slow Theta during the RSEO condition presented with a similar profile as the eyes closed condition with a global decrease in spectral power most pronounced along the midline (Figure 3). All central channels along with parietal channels P4 and Pz, and frontal midline Fz were significantly decreased during the THC visit compared to placebo before FDR correction.

A similar pattern of diminished power during the THC visit appeared in the fast Alpha frequency band that were mainly centered over midline central and parieto-occipital cortices (Figure 3 and Table 2). Spectral power from the two visits were also significantly different at the left temporal channel, T3 and left frontal channel, F7. In both resting state conditions, cannabis intoxication was associated with decreased spectral power relative to placebo at lower frequencies (1–12 Hz) and greater power at faster frequencies, 30–40 Hz (Supplementary Figures A1, A2).

**TABLE 2:** Paired *t*-test statistics for absolute spectral power during RSEO comparing the THC and placebo condition.

Frequency band	Channel	*t*-statistic	d.f.	*p*-value	THC visit	Placebo visit	CI (lower, upper)
Slow Theta (3–5 Hz)	C3	−2.99	9	0.0152	1.21 ± 0.53	1.675 ± 0.43	−0.818, −0.113
	C4	−2.79	9	0.021	1.17 ± 0.59	1.829 ± 0.63	−1.193, −0.125
	Cz	−2.63	9	0.0272	2.742 ± 1.14	3.635 ± 1.14	−1.66, −0.126
	Fz	−2.45	9	0.037	2.03 ± 0.76	2.864 ± 0.99	−1.606, −0.0626
	P4	−2.32	9	0.0457	1.076 ± 0.53	1.62 ± 0.62	−1.075, −0.013
	Pz	−2.7	9	0.0245	1.749 ± 0.63	2.5 ± 0.8	−1.389, −0.122
Fast Alpha (10–12 Hz)	Cz	−2.57	9	0.0302	1.497 ± 0.7	2.516 ± 1.5	−1.915, −0.122
	F7	−2.69	9	0.025	0.511 ± 0.28	0.854 ± 0.44	−0.632, −0.054
	PO	−2.4	9	0.0398	2.072 ± 1.41	3.377 ± 2.81	−2.534, −0.0755
	Pz	−2.64	9	0.0271	1.812 ± 1.29	2.997 ± 2.32	−2.203, −0.168
	T3	−2.57	9	0.0301	0.468 ± 0.36	0.746 ± 0.47	−0.523, −0.033

*d.f., degrees of freedom; CI, confidence interval.*

### Three-Choice Vigilance Task Event-Related Potentials

**FIGURE 4:**
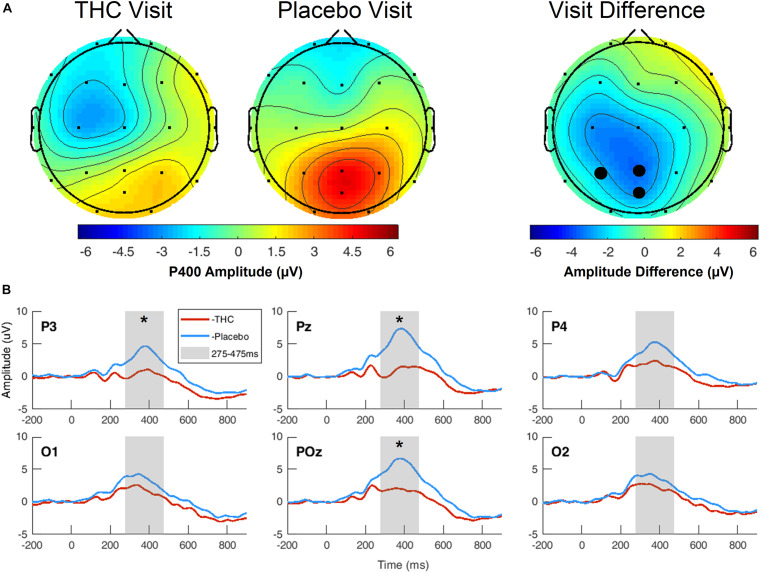
Three-choice vigilance task, Target stimulus at P400 component. **(A)** Amplitude of P400 peak following Target stimulus elicited an attenuated response during THC visit across parietal and occipital channels. Topographic maps represent average amplitudes between 275 and 475 ms post-stimulus. Difference between visits was significant at P3, Pz, and POz. **(B)** Average ERP traces for adjacent posterior channels reveal flattened P400 peaks associated with THC visit. Asterisk at P3 indicates significance at α = 0.05. Gray boxes depict P400 time range. THC visit, red line; Placebo visit, blue line.

During the THC visit, subjects exhibited lower amplitudes in the P400 component elicited by the Target stimulus. This reduction was localized to parietal and occipital regions (Figure 4A and Supplementary Figure B1). The differences in amplitudes between the two visits were significant at parietal channels P3, Pz, and POz before FDR correction (Table 3). This amplitude decrease during the THC visit was the result of a flattening of the P400 peak in comparison to the placebo visit (Figure 4B).

**TABLE 3:** Event-related potentials, significant comparisons between THC and placebo visit.

3-Choice vigilance task (3CVT)									
Stimulus	Component	Channel	*t*-statistic	d.f.	*p*-value	THC visit	Placebo visit	CI (lower, upper)
Target	P400	P3	−3.1	7	0.0174	0.44 ± 2.29	2.879 ± 1.86	−3.37, −0.45
		Pz	−2.99	8	0.0174	1.228 ± 3.7	4.744 ± 3.81	−6.23, −0.8
		POz	−2.67	8	0.0282	1.736 ± 3.46	4.743 ± 3.13	−5.6, −0.41

**Verbal memory scan (VMS)**									
Target	LPP	Fp	4.13	6	0.0062	0.12 ± 3.07	−5.8 ± 3.91	2.41, 9.42
		O1	−2.97	6	0.0248	0.088 ± 3.54	2.091 ± 3.06	−3.65, −0.36
		O2	−4.38	6	0.0047	0.702 ± 2.94	2.193 ± 2.37	−2.32, −0.66
NonTarget	LPP	Fp1	3.17	7	0.0157	0.799 ± 6.1	4.912 ± 5.69	1.45, 9.97
		POz	2.67	6	0.0372	2.846 ± 4.19	4.946 ± 2.99	−4.14, −0.18

*d.f., degrees of freedom; CI, confidence interval.*

### Verbal Memory Scan Event-Related Potentials

**FIGURE 5:**
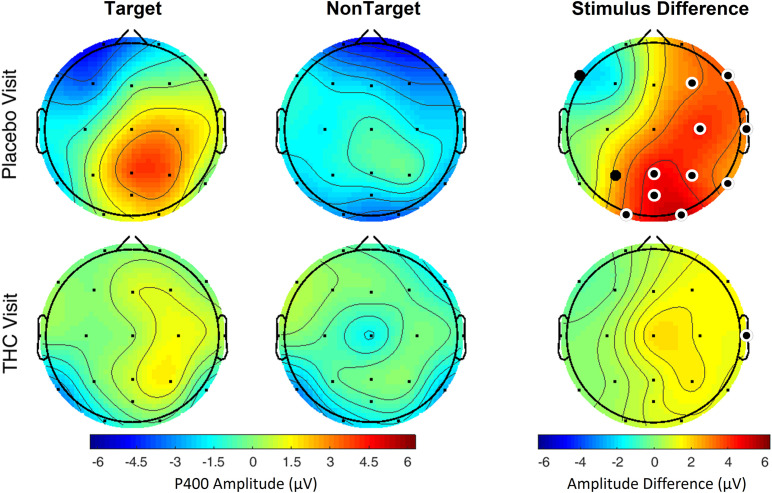
Verbal memory scan, P400 by visit and stimulus. Robust amplitude differences between Target and NonTarget stimuli seen during the placebo visit were significantly reduced during THC visit. Filled black circles mark significant channels before FDR correction for multiple comparisons; white rings indicate channels that remained significant after FDR correction for multiple comparisons.

Subjects’ P400 responses were different to the Target and NonTarget stimuli depending on visit. During the placebo visit, significant differences between Target and NonTarget were widespread across the right hemisphere (Figure 5) with Target words eliciting robust P400 peaks relative to NonTargets even after FDR correction (Table 4). This effect was attenuated considerably in the same subjects during the THC visit. While average P400 amplitudes were greater for Target stimuli than for NonTarget ones, significant differences between the two visits were limited to right temporal channel T4. In both visits, maximum P400 amplitudes were largely concentrated in the right hemisphere. ERP traces from the VMS task reveal the dynamics of the diminished response to Target words for subjects during the THC visit (Supplementary Figure C1).

**TABLE 4:** Event-related potentials for verbal memory scan task, significant comparisons between Target and NonTarget Stimuli.

Verbal memory scan (VMS)									
Visit	Component	Channel	*t*-statistic	d.f.	*p*-value	Target (μV)	NonTarget (μV)	CI (lower, upper)
Placebo	P400	C4	4.74	6	0.0032	2.377 ± 2.95	−1.816 ± 3.12	1.07, 7.32^‡^
		F4	3.85	5	0.012	−0.065 ± 2.15	−3.129 ± 3.42	0.022, 6.02^‡^
		F7	−2.75	5	0.0404	−3.566 ± 2.16	−1.426 ± 2.38	−5.59, −0.19
		F8	6.71	6	0.0005	−0.038 ± 2.75	−3.558 ± 3.45	1.67, 5.37^‡^
		O1	3.53	6	0.0123	0.577 ± 4.27	−2.977 ± 3.89	4.9e−15, 7.11^‡^
		O2	6.31	6	0.0007	1.858 ± 3.29	−3.52 ± 2.45	2.37, 8.39^‡^
		P3	2.62	6	0.0394	0.875 ± 2.59	−1.534 ± 3.64	0.16, 4.66
		P4	4.1	6	0.0063	3.36 ± 3.58	−0.556 ± 2.19	0.55, 7.28^‡^
		Poz	3.86	6	0.0083	2.473 ± 4.31	−2.32 ± 3.27	0.41, 9.17^‡^
		Pz	3.93	6	0.0077	3.722 ± 4.43	−0.968 ± 3.08	0.47, 8.91^‡^
		T4	8.11	6	0.0002	0.972 ± 2.26	−2.072 ± 1.52	1.72, 4.37^‡^
		T6	12.09	6	0.00001	−0.154 ± 1.55	−2.895 ± 1.16	1.94, 3.54^‡^
THC	P400	T4	3.6	6	0.0114	0.566 ± 2.03	−0.771 ± 1.8	0.43, 2.25
Placebo	LPP	C4	3.0	6	0.024	4.025 ± 4.43	1.155 ± 4.18	0.53, 5.21
		F4	5.1	5	0.0038	1.316 ± 5.34	−3.175 ± 6.44	0.11, 7.1^‡^
		F8	4.64	6	0.0035	4.325 ± 7.58	−2.976 ± 5.68	0.29, 14.31^‡^
		Fp2	2.72	5	0.0416	−2.577 ± 5.31	−6.537 ± 7.15	0.18, 6.18
		T4	4.46	6	0.0043	3.714 ± 4.04	−0.368 ± 2.43	8.9e−16, 8.16^‡^
		T6	3.55	6	0.0121	0.466 ± 1.89	−1.486 ± 1.74	0.61, 3.3

*^‡^Significant after FDR correction for multiple comparisons; d.f., degrees of freedom; CI, confidence interval.*

**FIGURE 6:**
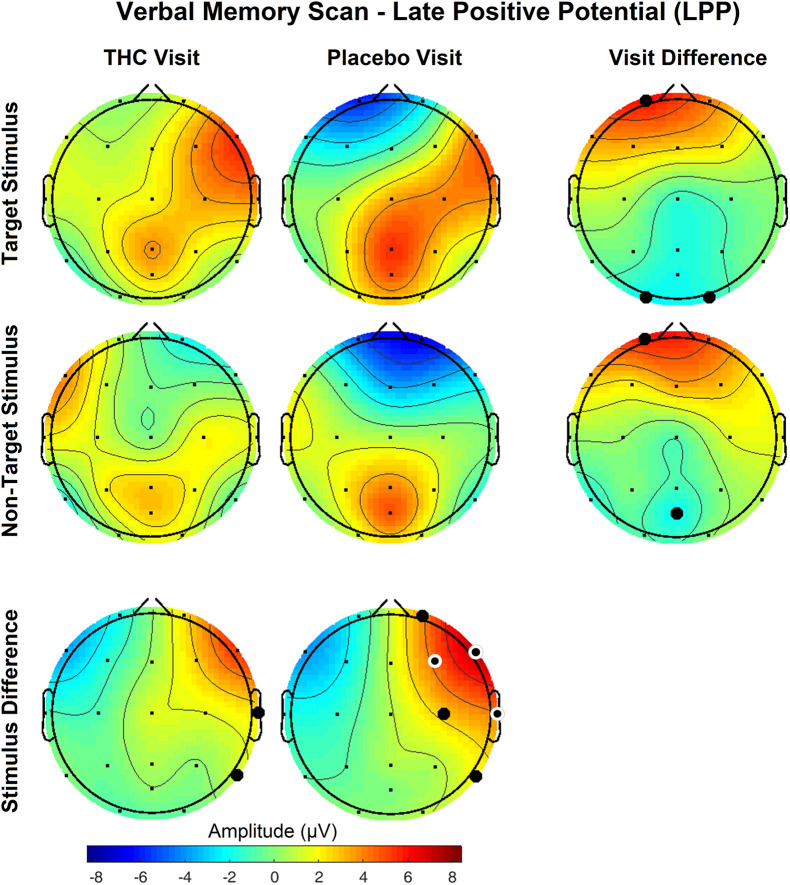
Verbal memory scan, late positive potential by visit and stimulus. Significant differences in LPP were found both between visits and between stimuli. Effects of visit read horizontally across figure; effects of stimulus are arranged vertically. Differences between Target and NonTarget stimuli were significantly elevated over right frontal and central channels during placebo visit. These differences all but disappeared during THC visit. Filled black circles mark significant channels before FDR correction for multiple comparisons; white rings indicate channels that remained significant after FDR correction for multiple comparisons. Numerical results of statistical analysis in Tables 3, 4.

The disparity between Target and NonTarget stimuli by visit seen in the P400 results extended to VMS LPP. Differences in the effect of the Target and NonTarget stimuli on LPP were significant in right frontal, central, and temporal channels during the placebo visit (Figure 6). This stimulus-related effect was weaker during the THC visit. Between-visit comparisons of LPP revealed significantly elevated frontal LPP amplitudes in subjects during their THC visit. LPP was significantly increased at Fp1 during subjects’ THC visit in both Target and NonTarget trials (Table 3). The elevated frontal LPP amplitudes associated with the THC visit were accompanied by significant amplitude decreases in parieto-occipital LPP relative to results from the placebo visit.

### Behavioral Performance on the Three-Choice Vigilance Task and Verbal Memory Scan

Performance during the 3CVT and VMS tasks was measured by the mean reaction time (RT), percentage of correct responses, and F-measure, which is a measure of performance that combines the harmonic mean of normalized accuracy and reaction time (Stikic et al., 2011). None of these performance measures were significantly different between the THC and placebo visits (Table 5).

**TABLE 5:** Reaction time (RT), percent correct, and F-measure for the 3CVT and VMS tasks, presented with means and standard deviations.

Task	Measure	THC visit	Placebo visit	Difference
3CVT	Reaction time (s)	0.85 ± 0.13	0.82 ± 0.13	0.03 (*p* = 0.30, df = 8)
	Percent correct	92.46 ± 12.87	96.22 ± 4.35	−3.75 (*p* = 0.25, df = 8)
	F-measure	0.68 ± 0.09	0.71 ± 0.09	−0.03 (*p* = 0.04, df = 8)
VMS	Reaction time (s)	1.09 ± 0.21	1.09 ± 0.16	0.0
	Percent correct	62.6 ± 29.83	85.65 ± 11.14	−23.05 (*p* = 0.06, df = 8)
	F-measure	0.47 ± 0.17	0.49 ± 0.14	−0.02 (*p* = 0.69, df = 8)

### Inter-Channel Coherence

**FIGURE 7:**
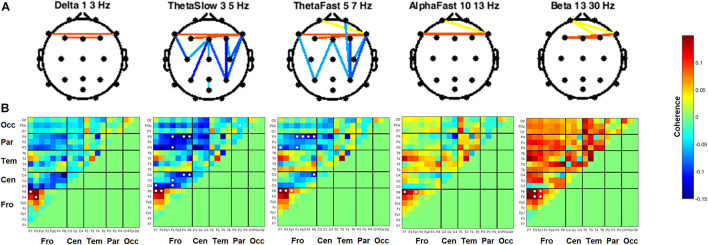
Inter-channel coherence differences between visits. Resting state eyes closed. THC visit was associated with increased coherence between inter-hemispheric frontal channels and coherence reductions along fronto-posterior axis. **(A)** Topographic maps by frequency band. **(B)** Accompanying heat maps of coherence by frequency band for all channel pairs. Significant pairs at α = 0.05 marked with white circles. Duplicate channel pair data below diagonal in heat map matrices have been set to zero (green) to facilitate visualization of results. Channels grouped together by region. Occ, occipital channels; Par, parietal channels; Tem, temporal channels; Cen, central channels; Fro, frontal channels.

Coherence between all pairs of EEG channels was calculated for both resting state conditions. During RSEC, subjects exhibited greater inter-hemispheric coherence between frontal channels during the THC visit compared to the placebo visit (Figure 7A). These THC-associated increases in frontal coherence were widespread across the frequency domain including the Delta (1–3 Hz), slow and fast Theta (3–7 Hz), fast Alpha (10–13 Hz), and Beta (13–30 Hz) frequency bands. This elevated frontal coherence during the THC visit was accompanied by reduced coherence in slow and fast Theta between channels along the anterior–posterior axis (Table 6).

**TABLE 6:** Coherence statistics for resting state eyes closed.

Frequency	ch1	ch2	*p*-value	*t*-statistic	d.f.	THC visit	Placebo visit	Difference	CI (lower, upper)
Delta	F3	F4	0.0046	4.101	7	0.778 ± 0.033	0.615 ± 0.032	0.172	0.07, 0.27
	F7	F8	0.0049	4.049	7	0.784 ± 0.036	0.621 ± 0.055	0.176	0.07, 0.28
Slow Theta	F4	P4	0.0076	−3.703	7	0.248 ± 0.03	0.373 ± 0.024	−0.159	−0.26, −0.06
	P3	Fz	0.0079	−3.679	7	0.207 ± 0.03	0.35 ± 0.025	−0.154	−0.25, −0.06
	F8	P4	0.0098	−3.513	7	0.205 ± 0.028	0.316 ± 0.028	−0.146	−0.24, −0.05
	Fz	P4	0.0046	−4.087	7	0.227 ± 0.025	0.35 ± 0.019	−0.135	−0.21, −0.06
	F8	C4	0.0052	−3.993	7	0.596 ± 0.034	0.704 ± 0.034	−0.133	−0.21, −0.05
	F7	C3	0.0006	−5.926	7	0.598 ± 0.034	0.659 ± 0.03	−0.122	−0.17, −0.7
	F4	C4	0.009	−3.575	7	0.724 ± 0.033	0.79 ± 0.02	−0.108	−0.18, −0.04
	Fz	C3	0.0004	−6.313	7	0.612 ± 0.024	0.68 ± 0.025	−0.105	−0.14, −0.07
	Fz	Cz	0.0071	−3.756	7	0.737 ± 0.038	0.799 ± 0.018	−0.09	−0.15, −0.03
	Pz	POz	0.009	−3.58	7	0.823 ± 0.023	0.882 ± 0.014	−0.042	−0.07, −0.01
	F8	F3	0.0077	3.698	7	0.735 ± 0.039	0.59 ± 0.041	0.17	0.06, 0.28
	F7	F8	0.0045	4.121	7	0.772 ± 0.041	0.595 ± 0.051	0.186	0.08, 0.29
Fast Theta	F8	P4	0.0024	−4.62	7	0.205 ± 0.031	0.314 ± 0.012	−0.123	−0.19, −0.06
	F4	P4	0.0047	−4.077	7	0.235 ± 0.03	0.349 ± 0.013	−0.121	−0.19, −0.05
	F8	C4	0.001	−5.436	7	0.641 ± 0.041	0.739 ± 0.025	−0.107	−0.15, −0.06
	Fp2	P4	0.0089	−3.584	7	0.177 ± 0.023	0.263 ± 0.01	−0.096	−0.16, −0.03
	Fz	P4	0.0075	−3.711	7	0.21 ± 0.026	0.307 ± 0.014	−0.094	−0.15, −0.03
	P3	Fz	0.009	−3.578	7	0.204 ± 0.033	0.301 ± 0.024	−0.085	−0.14, −0.03
	F7	P3	0.0065	−3.828	7	0.191 ± 0.032	0.282 ± 0.027	−0.079	−0.13, −0.03
	Fp1	F8	0.0034	4.341	7	0.877 ± 0.017	0.791 ± 0.028	0.082	0.04, 0.13
	F8	F3	0.0072	3.75	7	0.788 ± 0.029	0.636 ± 0.039	0.17	0.06, 0.28
	F7	F8	0.0041	4.182	7	0.813 ± 0.031	0.636 ± 0.045	0.181	0.08, 0.28
Slow Alpha	F4	O2	0.0005	6.045	7	0.271 ± 0.037	0.202 ± 0.017	0.027	0.02, 0.04
	F8	O2	0.0046	4.089	7	0.276 ± 0.033	0.205 ± 0.019	0.036	0.02, 0.06
	Fp2	O2	0.0093	3.557	7	0.304 ± 0.036	0.22 ± 0.02	0.044	0.01, 0.07
Fast Alpha	Fp1	F8	0.0096	3.534	7	0.877 ± 0.017	0.799 ± 0.023	0.086	0.03, 0.14
	F7	F8	0.0098	3.514	7	0.764 ± 0.039	0.622 ± 0.042	0.159	0.05, 0.27
Beta	Fp1	F8	0.0018	4.872	7	0.817 ± 0.025	0.718 ± 0.041	0.113	0.06, 0.17
	Fp1	F4	0.0085	3.618	7	0.784 ± 0.036	0.677 ± 0.027	0.117	0.04, 0.19
	F3	F4	0.0081	3.66	7	0.677 ± 0.051	0.535 ± 0.027	0.195	0.07, 0.32
	F8	F3	0.0054	3.971	7	0.667 ± 0.046	0.531 ± 0.043	0.199	0.08, 0.32
Gamma	Fp1	F8	0.0073	3.74	7	0.763 ± 0.036	0.658 ± 0.056	0.119	0.04, 0.19

*d.f., degrees of freedom; CI, confidence interval.*

**FIGURE 8:**
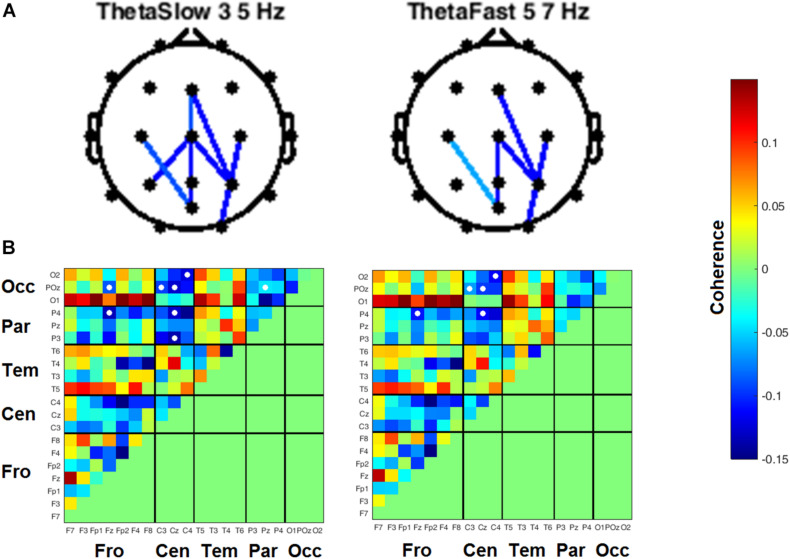
Inter-channel coherence differences between visits, resting state eyes open. Reduction of coherence between anterior and posterior regions during THC visit. **(A)** Topographic maps of channel pairs by frequency band significant at α = 0.05. **(B)** Accompanying heat maps of coherence by frequency band for all channel pairs. Significant pairs marked with white circles. Channels grouped by region for easier comparisons. Occ, occipital channels; Par, parietal channels; Tem, temporal channels; Cen, central channels; Fro, frontal channels.

Heat maps depicting average coherence for all channel pairs reveal a pattern of decreases in Theta frequency bands across all fronto-parietal, fronto-central, and centro-parietal channel pairs, regions where significant differences between visits were found (Figure 7B). In contrast to the diminished coherence in slower frequency bands, we found widespread increases in Beta band coherence between most channel pairs, albeit significant only at fronto-frontal pairs (Figure 7B and Table 6). While increased frontal coherence was only present under the eyes closed resting state condition, the reduced coherence between fronto-posterior and centro-posterior channels in the theta frequency range persisted in both eyes open and eyes closed resting state conditions (Figure 8 and Table 7).

**TABLE 7:** Coherence statistics for resting state eyes open.

Frequency	ch1	ch2	*p*-value	*t*-statistic	d.f.	THC visit	Placebo visit	Difference	CI (lower, upper)
Delta	T6	P3	0.0083	3.64	7	0.251 ± 0.035	0.182 ± 0.041	0.091	0.03, 0.15
Slow Theta	Pz	POz	0.0017	−4.904	7	0.829 ± 0.022	0.869 ± 0.019	−0.044	−0.07, −0.02
	Fz	POz	0.009	−3.578	7	0.198 ± 0.035	0.269 ± 0.031	−0.087	−0.14, −0.03
	POz	C3	0.0085	−3.618	7	0.301 ± 0.043	0.388 ± 0.036	−0.095	−0.16, −0.03
	C4	O2	0.0019	−4.824	7	0.221 ± 0.043	0.295 ± 0.033	−0.122	−0.18, −0.06
	POz	Cz	0.0013	−5.174	7	0.362 ± 0.043	0.459 ± 0.033	−0.127	−0.19, −0.07
	P4	Cz	0.0015	−5.025	7	0.406 ± 0.041	0.483 ± 0.037	−0.129	−0.19, −0.07
	Fz	P4	0.002	−4.8	7	0.235 ± 0.042	0.327 ± 0.034	−0.132	−0.2, −0.07
	P3	Cz	0.0074	−3.724	7	0.39 ± 0.043	0.503 ± 0.029	−0.137	−0.22, −0.05
Fast Theta	POz	C3	0.0046	−4.095	7	0.307 ± 0.044	0.37 ± 0.033	−0.066	−0.1, −0.03
	POz	Cz	0.0054	−3.973	7	0.362 ± 0.043	0.436 ± 0.029	−0.095	−0.15, −0.04
	Fz	P4	0.0057	−3.924	7	0.215 ± 0.036	0.292 ± 0.028	−0.107	−0.17, −0.04
	P4	Cz	0.006	−3.883	7	0.386 ± 0.04	0.456 ± 0.031	−0.112	−0.18, −0.04
	C4	O2	0.0097	−3.523	7	0.219 ± 0.037	0.294 ± 0.034	−0.115	−0.19, −0.04
Slow Alpha	F7	O1	0.009	3.575	7	0.257 ± 0.037	0.151 ± 0.015	0.114	0.04, 0.19
	F7	POz	0.0095	3.538	7	0.201 ± 0.02	0.136 ± 0.013	0.083	0.03, 0.14
	F7	P4	0.0081	3.654	7	0.202 ± 0.019	0.148 ± 0.015	0.062	0.02, 0.1
	T6	C3	0.0064	3.833	7	0.185 ± 0.02	0.155 ± 0.022	0.059	0.02, 0.1
	T6	F3	0.0002	6.913	7	0.175 ± 0.016	0.141 ± 0.017	0.053	0.04, 0.07
	Fp1	T6	0.0054	3.97	7	0.16 ± 0.012	0.139 ± 0.016	0.044	0.02, 0.07
	T6	Fz	0.0028	4.509	7	0.156 ± 0.013	0.126 ± 0.011	0.034	0.02, 0.05
Fast Alpha	F3	O2	0.0029	4.465	7	0.168 ± 0.016	0.123 ± 0.007	0.052	0.02, 0.08
	T6	F3	0.0084	3.63	7	0.168 ± 0.016	0.155 ± 0.02	0.043	0.02, 0.07
	T6	C3	0.0097	3.53	7	0.177 ± 0.016	0.151 ± 0.016	0.052	0.02, 0.09

*d.f., degrees of freedom; CI, confidence interval.*

### Changes in Heart Rate During All Tasks

Heart rates were significantly elevated during THC visit relative to placebo for all tasks (Table 8). This effect was highest during the resting tasks, with an observed increase of 24 BPM during RSEO and 30 BPM during RSEC. There was no significant difference in heart rate variability (LF/HF ratio) between the placebo and THC visits during any task.

**TABLE 8:** Mean heart rate for each task in each condition.

Task	THC	Placebo	Difference
RSEC	95.3 ± 14.7	64.6 ± 11.4	30.62 (*p* = 0.001, df = 7)
RSEO	88.6 ± 13.3	65.0 ± 10.4	23.59 (*p* = 0.009, df = 7)
3CVT	80.1 ± 13.2	68.4 ± 10.5	11.66 (*p* = 0.003, df = 8
VMS	88.6 ± 12.8	69.7 ± 9.5	18.81 (*p* = 0.001, df = 8)

## Discussion

The findings of this investigation indicate that acute cannabis intoxication was associated with (1) decreased spectral power in the Theta frequency band under both eyes-open and eyes-closed resting state conditions with accompanying decreases in power in the Alpha frequency range during eyes-open, and increases in Gamma band power during the eyes-closed resting state task, (2) decreased P400 amplitudes from Target stimuli in both 3CVT and VMS tasks and (3) decreased LPP during the VMS task, (4) increased inter-hemispheric frontal coherence in Delta (1–3 Hz), Theta (3–7 Hz), fast Alpha (10–13 Hz), and Beta (13–30 Hz) bands, and (5) a decrease of Theta band coherence in channel pairs falling along anterior–posterior axis during the THC visit relative to placebo.

Converging evidence indicates that the Theta frequency range is mechanistically important to both endogenous and exogenous cannabinoid activity. Previous studies investigating the acute effects of cannabis intoxication on EEG activity during resting states have reported significant decreases in Theta power (Ilan et al., 2004; Böcker et al., 2009; Hart et al., 2010). Intravenously delivered Δ^9^-THC in quantities approximately equivalent to one cannabis cigarette has been shown to decrease both spectral power and coherence in Theta band across bi-frontal electrodes during the performance of an n-back working memory task (Morrison et al., 2011).

The diminution of Theta power during acute cannabis intoxication appears to be refractory in heavy cannabis users that are temporarily abstinent. Prashad et al. (2018) reported elevated spectral power in the Theta band of regular cannabis users that had been 24-h abstinent compared to controls that had used cannabis fewer than 5 instances in their lifetime. Subjects who smoked cannabis cigarettes also presented with reduced Theta power during a resting state task and exhibited ERPs with attenuated amplitudes in N100, P200, and P300 components during a working memory task (Ilan et al., 2004).

Interestingly, activity in the Theta frequency band appears to be specifically sensitive to genetic variability in the cannabinoid receptor 1 gene, CNR1, which is highly expressed in the brain and mediates the psychoactive effects of cannabis intoxication. Researchers gathered resting state EEG data in subjects genotyped for a polymorphism in the coding region of the CNR1 gene and found significant differences between A-allele carriers and G/G homozygotes that was specific to the theta band (Heitland et al., 2014). Cannabis use has also been associated with an elevated risk of psychosis in vulnerable individuals (Morrison et al., 2011), and clinical studies of schizophrenia patients have found evidence of decreased fronto-parietal coherence in the Theta band (Griesmayr et al., 2014). Our resting state results showing elevated Gamma and decreased Alpha power agree with earlier studies where cannabis use has previously been shown to increase power in Gamma band most prominently in posterior brain regions (Nottage et al., 2015) and to reduce Alpha power during cognitive and perceptual tasks (Jones and Stone, 1970) and in spatial working memory tasks (Hart et al., 2010).

In the crossover design we employed, we found evidence for reduced coherence between channels falling along the anterior–posterior axis in subjects during the THC visit (Figures 7, 8). Theta band activity across fronto-posterior cortical networks has previously been implicated in cannabis intoxication (Prashad et al., 2018; Rangel-Pacheco et al., 2020), and interactions between executive function and working memory (Sauseng et al., 2005; Griesmayr et al., 2014; Park et al., 2019). An fMRI-based repeated measures design revealed that functional connectivity between brain structures comprising the default mode network (DMN) was significantly reduced in subjects during their Δ^9^-THC dosing visit compared to scans taken when subjects were given placebo (Wall et al., 2019). The DMN is anatomically distributed between anterior and posterior brain regions, with the medial prefrontal cortex (MPC) comprising the anterior portion and a collection of neighboring posterior regions that include precuneus, posterior cingulate cortex, and angular gyrus. The Δ^9^-THC-disrupted connectivity between DMN brain regions is in alignment with the fronto-parietal and fronto-occipital channels reductions in coherence that we report here (Figures 7, 8).

In the current study, the reductions in fronto-posterior coherence were accompanied by increased inter-hemisphere frontal coherence during RSEC (Figure 7). Increasing cognitive control demands elicited greater functional connectivity between prefrontal and occipito-parietal cortices in 12-h abstinent heavy cannabis users compared to non-using controls (Harding et al., 2012). The authors suggest that this increase associated with elevated cognitive load reflects additional effort required by chronic cannabis users to maintain effective behavioral performance on the cognitive control task they employed. These results are in line with previous studies indicating some compensatory neuroadaptation providing tolerance to the adverse effects of cannabis intoxication on cognitive processes in habitual cannabis users (Hart et al., 2001, 2010; Ramaekers et al., 2009).

Event-related potentials) are scalp-recorded EEGs synchronized at the millisecond level to the presentation of auditory, visual, or somatosensory stimuli (Luck, 2014). ERPs appear to track with the flow of information from sensory processing and analysis to response. Early components reflect sensory processing of the characteristics of the stimuli but can be influenced by arousal and attention (Hillyard et al., 1973; Coles et al., 1985, 1995). The late ERP components are thought to reflect feature evaluation, memory matching, and processing speed (Hillyard and Kutas, 1983; Polich and Kok, 1995). P300 has been associated with information encoding, memory processes, attention, and retrieval (D’Souza et al., 2012). Shorter P300 latency and increased P300 amplitude reflect superior cognitive performance, while delayed P300 latency and reduced P300 amplitude tend to reflect cognitive impairment (Campbell et al., 1979; Polich et al., 1986; Polich and Kok, 1995). While we found no significant differences between the THC and placebo visits in performance on the 3CVT and VMS tasks (Table 5), our results indicate that ERP amplitudes are sensitive to the effects of cannabis intoxication (Figures 4, 5). These results are consistent with previous work showing Δ^9^-THC-associated drops in P300 amplitudes (Böcker et al., 2010). Cannabis exposure has also been shown to dose-dependently reduce the amplitude of the P300 in working memory tests, choice decision tasks, and in an auditory oddball paradigm (D’Souza et al., 2012).

While cannabis intoxication appears to affect the same brain regions regardless of frequency of use, the same does not seem to be true with respect to its effects on behavioral performance. Results from several studies suggest that heavy cannabis users appear less affected on task performance than infrequent users. One study (Hart et al., 2010) looked at EEG of daily cannabis smokers before and after smoking cannabis as they engaged with tests of immediate working memory (spatial *n*-back task) and delayed episodic memory in a word recognition task. Accuracy was not affected by cannabis; however, response times rose in episodic memory test. Response times also increased in a dose-dependent fashion during the working memory test. ERP amplitudes within 400–700 ms post-stimulus (word presentation) were significantly decreased in subjects after smoking cannabis. The amplitude diminution of this component was greatest in posterior EEG channels, and significantly less than amplitudes at frontal sites. Despite the minimal decrements to task performance in daily cannabis users, they exhibited similar neurophysiological profiles of intoxication in infrequent cannabis users.

Based on this study and existing literature on the effects of cannabis on brain functioning, it appears that while performance impairment may vary depending on how tolerant users are to the effects of cannabis on cognition, similar patterns of spectral power and EEG coherence may emerge in subjects independent of the frequency of cannabis use. In another study, both occasional and heavy cannabis users engaged with a series of tasks to assess hand–eye coordination (critical tracking task), divided attention capacity, and motor impulsivity (Ramaekers et al., 2009). Performance on the tracking and divided attention tasks was significantly compromised in occasional users, but evidence of impairment was absent in heavy cannabis users in all tasks except in the Stop-Signal Task, a test of motor impulsivity. Following the Δ^9^-THC dose, heavy cannabis users had greater difficulty suppressing responses following the “stop” signal and presented with slower reaction times than occasional users in the first hour of Δ^9^-THC intoxication. These results prompted the authors to point out that the selective impairment in heavy cannabis users was consistent with studies linking long-term drug use to impairments in behavioral inhibition.

## Conclusion

This study implemented an integrated platform combining neurocognitive tests of attention and verbal memory administered with concurrent EEG and ECG data acquisition to identify robust and quantifiable biomarkers of acute cannabis intoxication. Our findings replicate prior work and provide new insights into the effects that cannabis intoxication has on several cognitive processes including attention and verbal memory. These biomarkers may prove useful as predictors of impairment from acute cannabis intoxication during functional, real-world tasks.

## Data Availability Statement

The raw data supporting the conclusions of this article will be made available by the authors, without undue reservation.

## Ethics Statement

All protocols and corresponding human subject facing documents were approved by University of Iowa IRB and when they were approved, they were then submitted to National Institute on Drug Abuse (NIDA). The patients/participants provided their written informed consent to participate in this study.

## Author Contributions

CB and TB: conceptualization, resources, project administration, and funding acquisition. CB and AM: methodology. AM and CR: software and validation. CR, MS-K, and AM: formal analysis. JP, MM, GR, RS, and AF: investigation. CR: data curation, writing – review and editing, and visualization. CR, JP, and GR: writing – original draft preparation. CB, TB, and AM: supervision. All authors have read and agreed to the published version of the manuscript.

## Conflict of Interest

The authors declare that the research was conducted in the absence of any commercial or financial relationships that could be construed as a potential conflict of interest.

## Publisher’s Note

All claims expressed in this article are solely those of the authors and do not necessarily represent those of their affiliated organizations, or those of the publisher, the editors and the reviewers. Any product that may be evaluated in this article, or claim that may be made by its manufacturer, is not guaranteed or endorsed by the publisher.
