# Supplementary figures and images for: Alterations in Electroencephalography Theta as Candidate Biomarkers of Acute Cannabis Intoxication
Source: Front Neurosci. 2021 Oct 4;15:744762. doi: 10.3389/fnins.2021.744762 (PMC8520987; doi:10.3389/fnins.2021.744762)

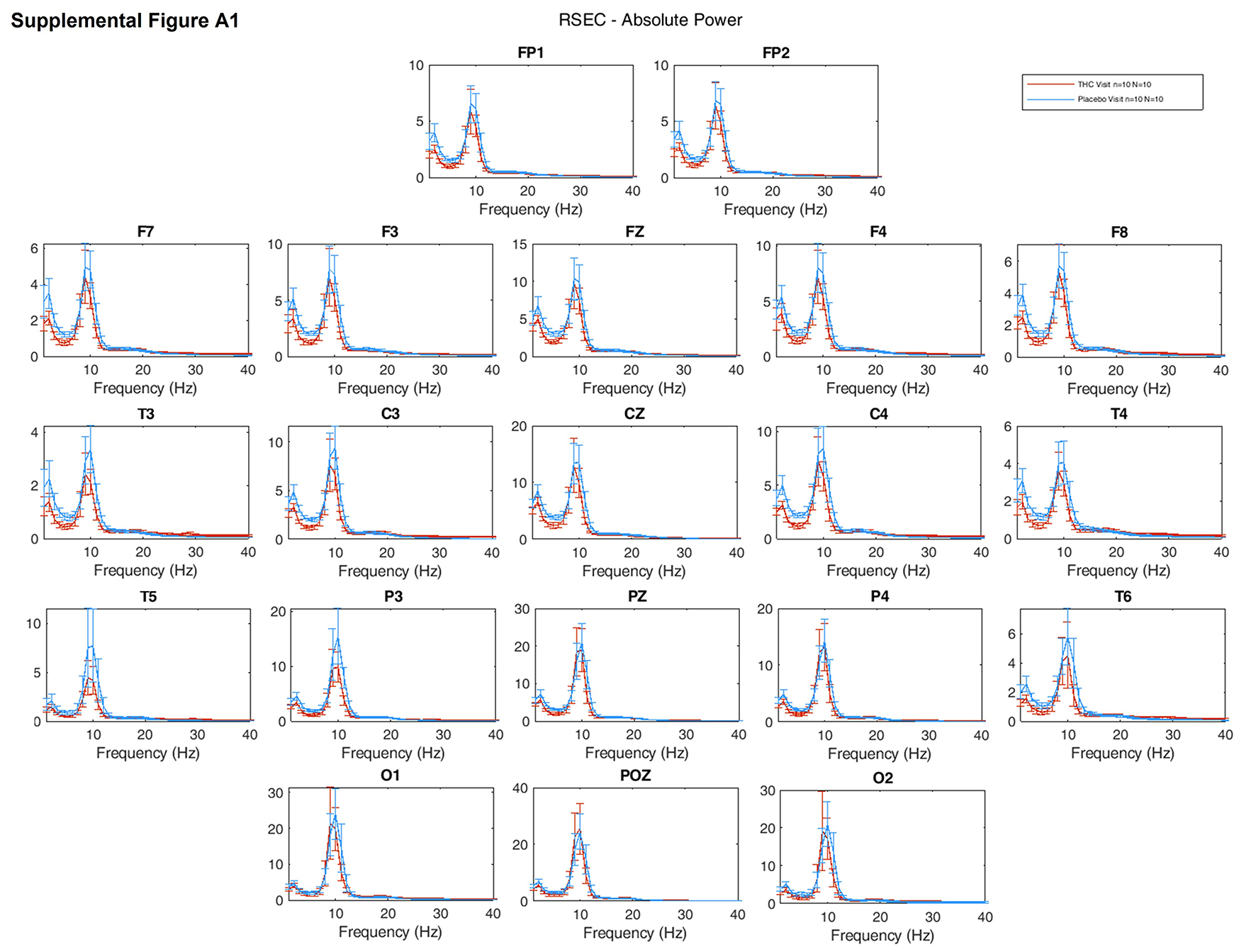

Supplement: Supplementary file 1 [file Image_1.jpeg]

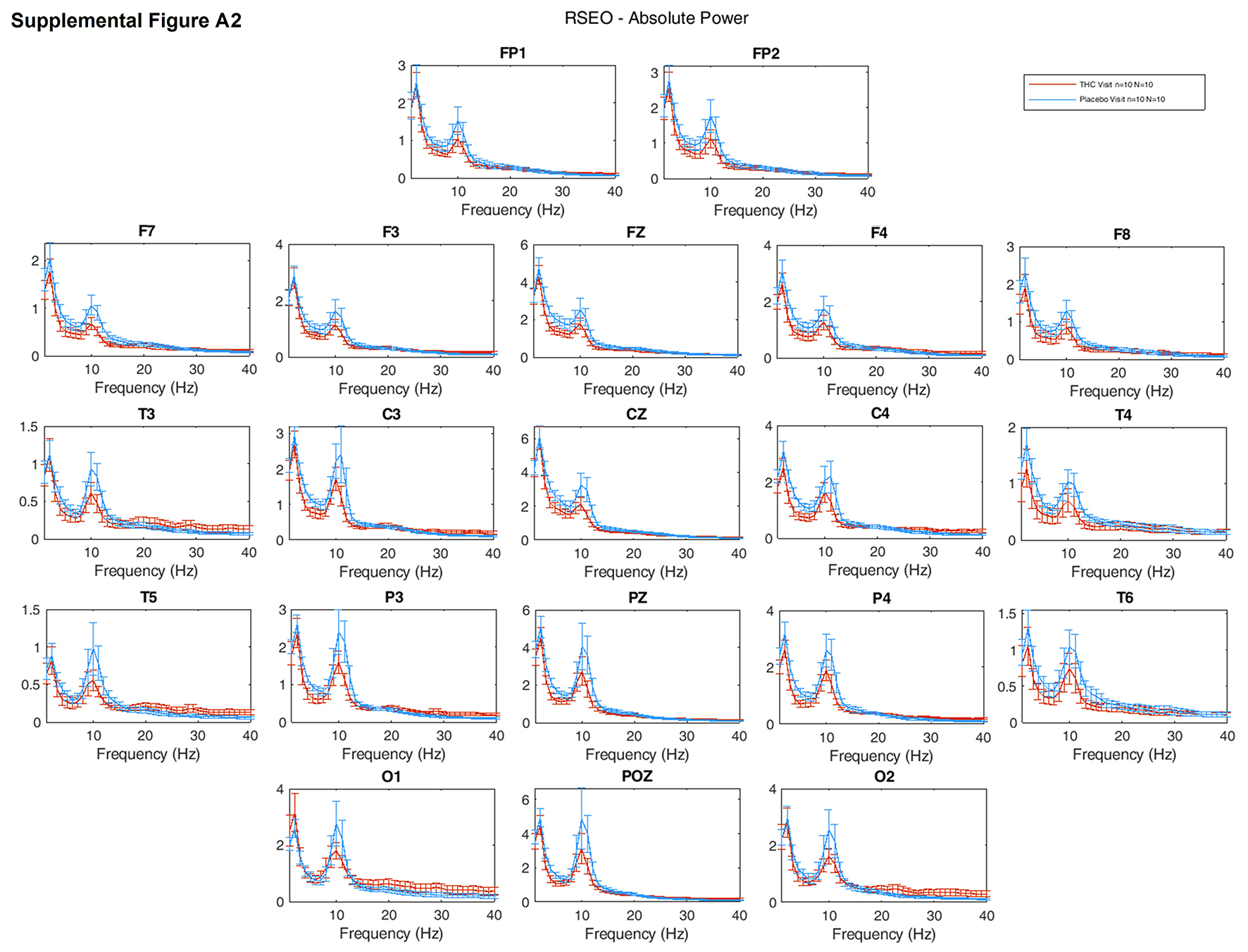

Supplement: Supplementary file 2 [file Image_2.jpeg]

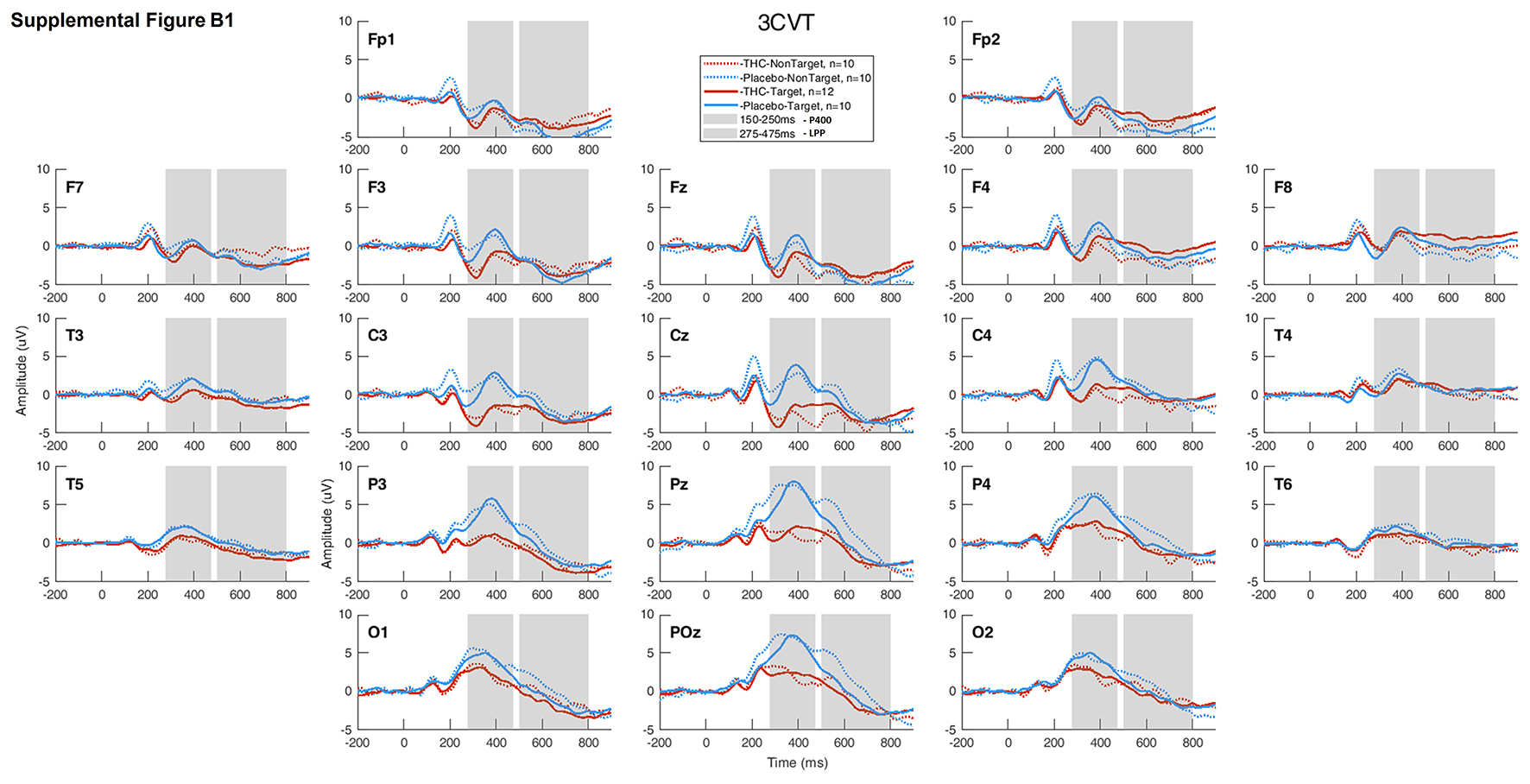

Supplement: Supplementary file 3 [file Image_3.jpeg]

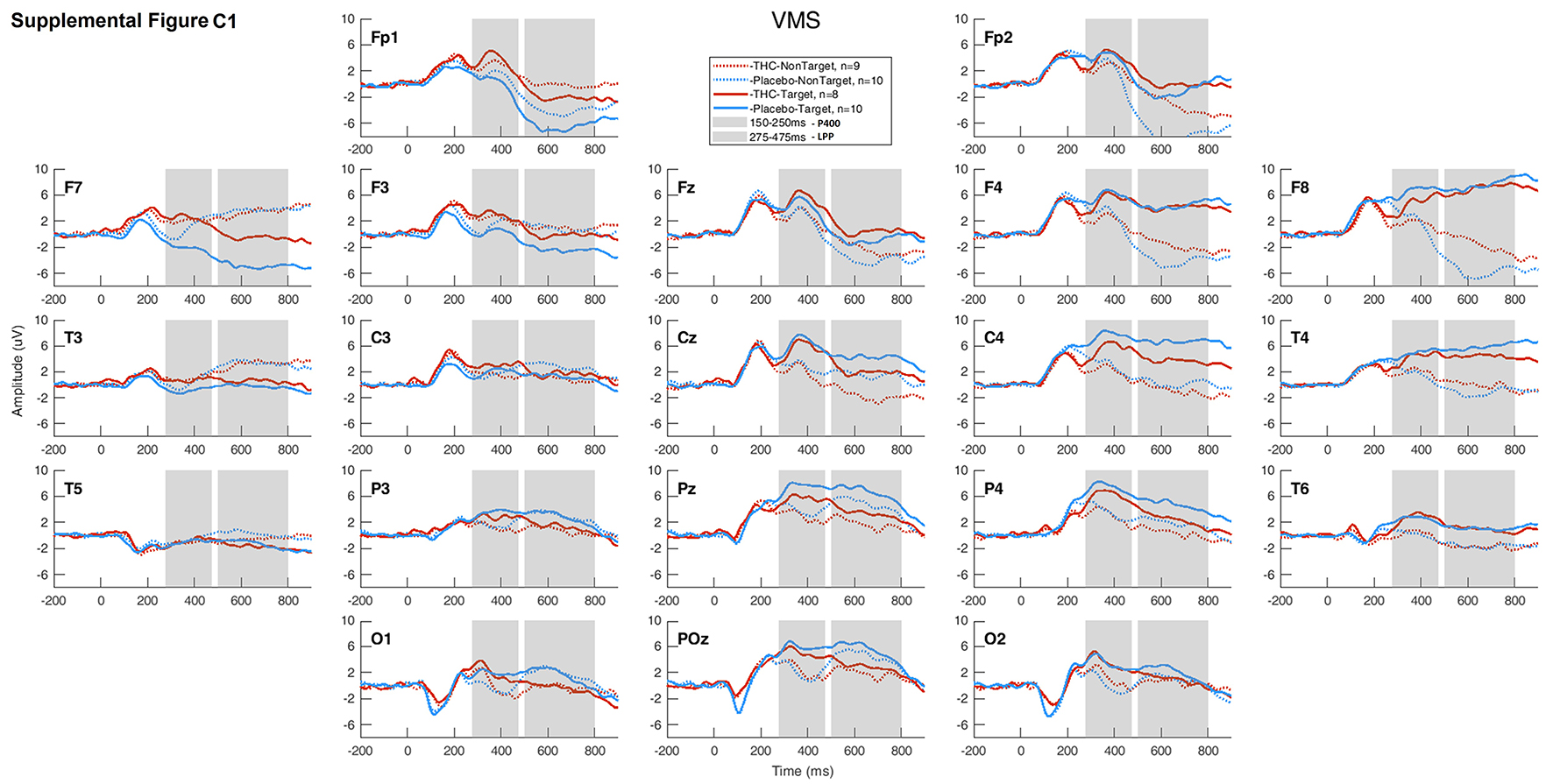

Supplement: Supplementary file 4 [file Image_4.jpeg]
